# Initial military training modulates serum fatty acid and amino acid metabolites

**DOI:** 10.14814/phy2.15385

**Published:** 2022-07-11

**Authors:** Jess A. Gwin, Adrienne Hatch‐McChesney, Kenneth P. Pitts, Rory P. O'Brien, Anthony J. Karis, Christopher T. Carrigan, James P. McClung, J. Philip Karl, Lee M. Margolis

**Affiliations:** ^1^ U.S. Army Research Institute of Environmental Medicine Natick Massachusetts USA; ^2^ U.S. Army Research Institute for the Behavioral and Social Sciences Fort Benning Georgia USA; ^3^ U.S. Army Maneuver Center of Excellence Fort Benning Georgia USA

**Keywords:** branched‐chain amino acids, fat mass, fatty acids, lean mass

## Abstract

Initial military training (IMT) results in increased fat‐free mass (FFM) and decreased fat mass (FM). The underlying metabolic adaptations facilitating changes in body composition during IMT are unknown. The objective of this study was to assess changes in body composition and the serum metabolome during 22‐week US Army IMT. Fifty‐four volunteers (mean ± SD; 22 ± 3 year; 24.6 ± 3.7 kg/m^2^) completed this longitudinal study. Body composition measurements (InBody 770) and blood samples were collected under fasting, rested conditions PRE and POST IMT. Global metabolite profiling was performed to identify metabolites involved in energy, carbohydrate, lipid, and protein metabolism (Metabolon, Inc.). There was no change in body mass (POST‐PRE; 0.4 ± 5.1 kg, *p =* 0.59), while FM decreased (−1.7 ± 3.5 kg, *p* < 0.01), and FFM increased (2.1 ± 2.8 kg, *p* < 0.01) POST compared to PRE IMT. Of 677 identified metabolites, 340 differed at POST compared to PRE (*p* < 0.05, *Q* < 0.10). The majority of these metabolites were related to fatty acid (73%) and amino acid (26%) metabolism. Increases were detected in 41% of branched‐chain amino acid metabolites, 53% of histidine metabolites, and 35% of urea cycle metabolites. Decreases were detected in 93% of long‐chain fatty acid metabolites, while 58% of primary bile acid metabolites increased. Increases in amino acid metabolites suggest higher rates of protein turnover, while changes in fatty acid metabolites indicate increased fat oxidation, which likely contribute changes in body composition during IMT. Overall, changes in metabolomics profiles provide insight into metabolic adaptions underlying changes in body composition during IMT.

## Graphical abstract

Results from the current study indicate robust changes in the serum metabolome following 22‐weeks of initial military training. Findings from this study give new insight into the anabolic response to initial military training, providing evidence that increases in BCAA metabolites in combination with increased circulating total and free testosterone may support accretion of fat free mass during initial military training.

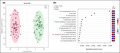

## 1 INTRODUCTION

Physical training including both aerobic and resistance exercise stimulates improvements in body composition over time. U.S. Army initial military training (IMT) represents the first component of occupational skill development and physical fitness training for Soldiers entering service. Previous studies demonstrate that the physical training component of IMT improves body composition by increasing fat‐free mass (FFM) and decreasing fat mass (FM), or a combination of the two (Foulis et al., 2021; Margolis et al., 2012; Nindl et al., 2011; Pasiakos et al., 2012; Williams, 2005). These changes are likely an adaptation to increased physical activity through both aerobic‐ and resistance‐based exercise. While the positive changes in body composition observed with increased physical training during IMT are well characterized, the underlying metabolic mechanisms accounting for these improvements have not been fully explored.

Global metabolomics analyses provide a comprehensive and sensitive assessment of hundreds to thousands of circulating metabolites, which enables high‐throughput identification of distinct, whole‐body metabolic signatures or biomarkers (Marciano & Snyder, 2019). Metabolomics analyses permit wide‐reaching mechanistic insight into metabolic regulation and adaptation to external stimuli such as changes in diet and physical activity. To our knowledge, the majority of metabolomic signature profiling associated with body composition changes has been conducted in relation to weight loss and weight management in unhealthy populations (Rangel‐Huerta et al., 2019). Cross‐sectional data suggest that metabolic profiles, particularly higher concentrations of circulating branched‐chain amino acid (BCAA) metabolites are associated with greater muscle mass (Jourdan et al., 2012; Lustgarten et al., 2013). However, the underlying metabolic adaptations facilitating changes in body composition in physically active, healthy populations, such as Soldiers participating in IMT, remains understudied.

The objective of this study was to assess changes in body composition and the serum metabolome using global metabolomics analyses in Soldiers following 22‐week US Army IMT. Based on previous work in a similar IMT environment (Margolis et al., 2012), we hypothesized that participants would increase FFM and decrease FM during the 22‐week IMT. Additionally, we hypothesized that there would be an increase in circulating BCAA metabolites to support increases in FFM and changes in lipid metabolites that would indicate increases in fat mobilization for fuel use to decrease FM.

## 2 METHODS

This observational, longitudinal study was conducted in US Army infantry recruits participating in IMT at Ft. Benning GA from February to July 2020. Only Army recruits aged 17 years and older participating in the training were eligible to participate in this study. Those who donated blood within the past 4 months at the time of study briefing were excluded. A total of 105 recruits were enrolled in the study. Of these participants, seven requested withdrawal prior to the start of the study, six requested withdrawal prior to the completion of the study, and 31 were withdrawn because they did not complete the training or were at sick call/quarantine at the time of data collection. Additionally, insufficient blood was available for metabolomics analysis in seven volunteers. As such, data for this investigation was analyzed on 54 volunteers (52 males, 2 females). This study was reviewed and approved by the US Army Medical Research and Development Command (Ft. Detrick) Institutional Review Board. All volunteers provided informed written consent.

## 3 TRAINING

IMT integrates physical fitness with military skills instruction in a tightly controlled, psychologically and physically challenging environment. For infantry Soldiers, IMT is a 22‐week training event, combining Basic Combat Training (BCT) and Advanced Individual Training (AIT). The BCT portion (10 weeks) of IMT focuses on aerobic (e.g., loaded road marches, sprinting, distance running) and strength training (i.e., calisthenics, pushups, sit‐ups) in the form of physical training sessions 4–6 days/week and military‐related activities (e.g., obstacle courses, weapons training, ruck marches, land navigation) along with didactic classroom instruction to learn basic soldiering skills (Knapik et al., 2007). The AIT portion is occupation‐specific training, providing specialized instruction in the Soldiers' career field. Physical training is maintained at 4–6 days/week, and military‐related activities and didactic classroom instruction are tailored to the Soldiers occupational specialty. Throughout the entirety of the 22‐week training, recruits reside in barracks; and receive three meals per day either from the dining facility or in the form of combat rations. For this study, all data were collected during weeks 0 (PRE) and 22 (POST) of the training.

### 3.1 Anthropometrics and body composition

Height was measured to the nearest cm using a stadiometer (Seca; Creative Health Products) at PRE. Body mass and composition were determined following a 10‐hr overnight fast using bioelectrical impedance (InBody 770; Inbody Co., LTD) to the nearest 0.1 kg (Gibson et al., 2008) at PRE and POST. For the measurement, volunteers were dressed in their physical training uniform, removed their socks, cleaned their feet and hands using cleansing wipes, stepped on the electrode of the foothold, and held the electrode handlebars.

Changes in body energy stores (∆ES) were used to estimate energy balance during IMT (Hoyt et al., 2006):

$$\begin{array}{c} \text{∆ES}=\left(\text{∆FM}\times 9.51\;\text{kcal}/g\right)\\ \;+\left[\text{∆FFM}\times \left(1-\mathrm{FFM}\;\text{hydration}\right)\times 4.40\;\text{kcal}/g\right], \end{array}$$

where ∆FM and ∆FFM are the change (POST–PRE) in FM and FFM, respectively, and (1 − FFM hydration) is the nonaqueous fraction of FFM, estimated as 0.73 (Siri, 1956).

### 3.2 Food frequency questionnaires

Energy and macronutrient intakes over the prior 3 months were assessed at PRE and POST from a self‐administered paper and pencil version of the 2014 Block Food Frequency Questionnaire (FFQ) (NutritionQuest, 2021). The Block FFQ is validated for use in the general US population (Block et al., 1990), and has previously been used to assess dietary intake during military trainings (Farina et al., 2017; Lutz et al., 2013, 2019). In the block FFQ, participants select the frequency at which they consumed food and beverage items listed on the FFQ and the usual portion size consumed. Pictures were provided to assist in the selection of portion sizes. Trained staff were available to answer questions and provide clarifications during the administration and collection of the FFQ. All dietary intake data was assessed as relative intake normalized to g/1000 kcal (Willett et al., 1997).

### 3.3 Blood analytes

Serum and plasma samples were collected by antecubital intravenous blood draws under resting conditions after a 10‐h overnight fast at PRE and POST. Samples were centrifuged at 3000 rpm at 4°C for 10 min. Serum and plasma were then stored at −80°C until analysis. Markers of androgen status were assessed due to their known anabolic relationship with FFM. Total testosterone, sex hormone–binding globulin, and luteinizing hormone were measured in duplicate with an advanced automated immunoassay instrument (Immulite 2000; Siemens Healthcare Diagnostic). Free testosterone was measured in duplicate using an ELISA assay kit (ALPCO; catalogue #11‐FTSHU‐E01). All samples were tested in duplicate and the coefficient of variation for sample duplicates were in the range of 0%–15%. Testing was repeated if the coefficient of variation for sample duplicates exceeded 15%. A weighted 5PL standard curve was determined and low and high controls within an acceptable range were included with each run.

### 3.4 Metabolomics

Global metabolomics profiling of serum samples was conducted using four separate methods: Two separate reverse phase (RP)/UPLC‐MS/MS methods with positive ion mode electrospray ionization (ESI), a RP/UPLC‐MS/MS method with negative ion mode ESI, and a HILIC/UPLCMS/ MS method with negative ion mode ESI (Metabolon Inc.). Technical replicates, blanks, internal standards, and several recovery standards were analyzed with experimental samples for quality control. Raw data were extracted, peaks were identified, and quality control was processed using proprietary hardware and software (Metabolon, Inc.). The relative quantitation values were determined using integrated peak areas (area under the curve). All samples were analyzed on an equivalency basis determined by volume.

Metabolites were identified by automated comparison of the ion features in the experimental samples to a references library of chemical standard entries that included retention time, molecular weight (m/z), preferred adducts, and in‐source fragments as well as associated MS spectra, and were curated by visual inspection for quality control using software developed at Metabolon (Metabolon Inc.) (Dehaven et al., 2010; Evans et al., 2009). The level of identification for the majority of the compounds detected meets the highest standard of metabolite identification according to the Metabolomics Standards Initiative (Sumner et al., 2007). Several types of controls were analyzed in concert with the experimental samples. A pooled matrix sample was generated by taking a small volume of each experimental sample to serve as a technical replicate throughout the data set. Extracted water samples served as process blanks. A cocktail of quality control standards that would not interfere with the measurement of endogenous compounds were spiked into every analyzed sample to allow instrument performance monitoring and aided chromatographic alignment. Instrument variability was determined by calculating the median relative standard deviation (RSD) for the standards that were added to each sample prior to injection into the mass spectrometers. Overall, process variability was determined by calculating the median RSD for all endogenous metabolites (i.e., non‐instrument standards) present in 100% of the pooled matrix samples.

### 3.5 Statistical analysis

Analyses were completed using R v4.0.3, SPSS v26 (IBM Analytics), ArrayStudio (Omicsoft Corp.), and MetaboAnalyst v.5.0 (Xia & Wishart, 2016). Before analysis of metabolomics data, any missing values were imputed using the minimum observed peak area for each compound. Peak areas for each metabolite were then normalized to set the mean equal to 0 and log10 transformed to meet model assumptions. Statistical analysis of metabolomics profiles was focused on metabolites relevant to energy and macronutrient metabolism. Orthogonal projections to latent structures discriminant analyses and random forest plots were conducted using MetaboAnalyst v.5.0 (Xia & Wishart, 2016) to assess the effect of time (PRE vs. POST) on changes in global metabolite profiles. Paired t‐tests were also used to determine differences between PRE and POST IMT for body mass and composition, energy and macronutrient intakes, blood analytes, and metabolites. Blood analytes were log10 transformed to meet model assumptions. To account for multiple comparisons, the Benjamini‐Hochberg method was used to estimate false discovery rate (*Q*‐value). Statistical significance was set at *p* < 0.05 and *Q* < 0.10.

## 4 RESULTS

**FIGURE 1:**
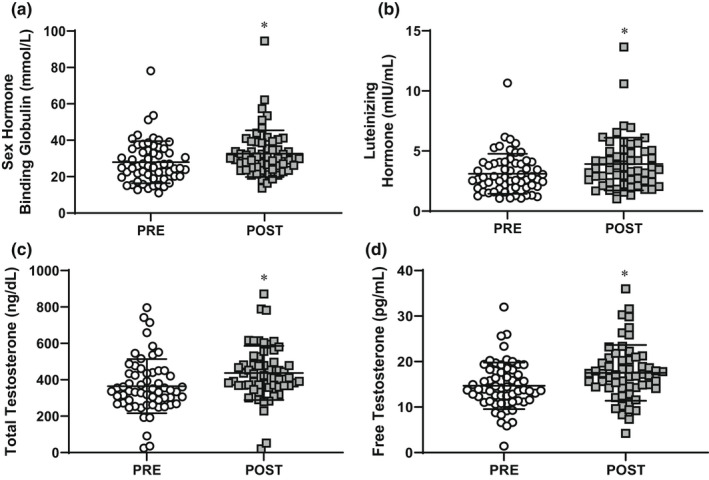
Mean ± SD log10 transformed sex‐hormone binding globulin (a), luteinizing hormone (b), total testosterone (c), and free testosterone (d) PRE and POST initial military training. *Different then PRE; paired *t*‐test, *p* < 0.05; *n* = 54 (52 males, 2 females).

There was no change in body mass (POST‐PRE ± SD; 0.4 ± 5.1 kg, *p* = 0.59) from PRE to POST (Table 1). There was a decrease in FM (−1.7 ± 3.5 kg, *p* < 0.01) and an increase in FFM (2.1 ± 2.8 kg, *p* < 0.01) POST compared to PRE. Dietary analysis was limited to a subset (n = 31) of volunteers who met the criterion of reporting plausible (i.e., reasonably based on body mass) energy intake at PRE and POST (Gwin et al., 2019; Pasiakos et al., 2012). The remainder of volunteers were considered implausible reporters or did not have complete data for both time points and were excluded from the analysis. Estimated energy balance for the entire training based on the change in energy stores was −87 ± 223 kcal. When normalized to 1000 kcal/day or expressed as percent of total energy intake, carbohydrate intake increased and fat intake decreased (*p* < 0.01) with no differences in energy or protein intake (*p* > 0.05) over the 3 mo prior to PRE relative to during IMT (Table 2). Concentrations of sex‐hormone binding globulin (4.9 ± 6.3 mmol/L), luteinizing hormone (0.7 ± 1.5 mIU/ml), total testosterone (66.9 ± 111.9 ng/dl), and free testosterone (2.8 ± 5.7 pg/ml) increased (*p* < 0.05) POST compared to PRE (Figure 1a–d).

**TABLE 1:** Body mass and composition PRE and POST initial military training

	PRE	POST	*p* value
Body mass (kg)	74.8 ± 13.0	75.1 ± 11.3	0.59
Fat mass (kg)	12.7 ± 7.2	11.0 ± 5.0	<0.01
Fat‐free mass (kg)	62.0 ± 9.1	64.1 ± 8.8	<0.01

*Note*: Values mean ± SD; paired *t*‐test, *p* < 0.05; *n* = 54 (52 males, 2 females).

**TABLE 2:** Relative energy and macronutrient intake PRE and POST initial military training

	PRE	POST	*p* value
Energy			
kcal/1000 kcal/day	2.3 ± 1.1	2.1 ± 0.9	0.54
Protein			
g/1000 kcal/day	40.6 ± 6.8	40.0 ± 6.1	0.71
% of total daily intake	16.2 ± 2.8	15.8 ± 2.6	0.63
Carbohydrate			
g/1000 kcal/day	109.6 ± 12.0	126.0 ± 18.0	<0.01
% of total daily intake	43.9 ± 4.8	50.4 ± 7.1	<0.01
Fat			
g/1000 kcal/day	43.9 ± 5.9	38.3 ± 5.3	<0.01
% of total daily intake	39.5 ± 5.3	34.5 ± 4.8	<0.01

*Note*: Values mean ± *SD*; paired *t*‐test, *p* < 0.05; *n* = 31 (29 males, 2 females).

**FIGURE 2:**
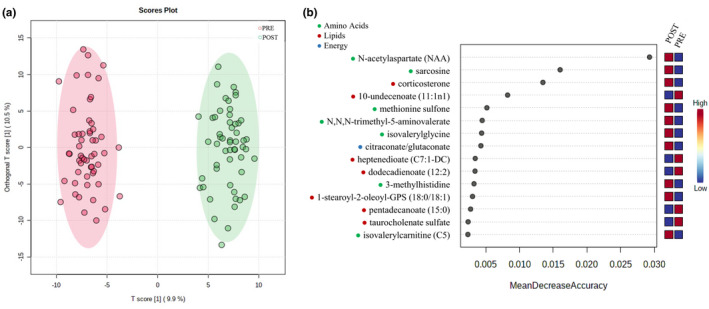
Orthogonal projections to latent structures discriminant analysis score plot for all metabolite features samples based on subject and time (PRE and POST) (a). Random forest plot depicting 15 identified metabolites that were 96% accurate in predicting time point (b); *n* = 54 (52 males, 2 females).

In total, 677 metabolites within pathways of amino acid, lipid, carbohydrate, and energy metabolism were identified, and 340 of those were significantly different (*p* < 0.05, *Q* < 0.10) at POST compared to PRE. The majority of the significantly different metabolites were related to fatty acid (73%) and amino acid (26%) metabolism. Orthogonal projections to latent structures discriminant analysis demonstrated a clear separation in metabolite profiles between PRE and POST (Figure 2a **)**. Random forest plot analysis identified 15 metabolites that were 96% accurate in predicting time point (Figure 2b). Eight metabolites had a ≥ 2 fold increase, and 22 metabolites had a ≥ 2 fold decrease at POST relative to PRE (*p* < 0.05, *Q* < 0.10; Table 3). These metabolites belonged to sub‐pathways for BCAA, histidine, long‐chain fatty acid, dicarboxylate fatty acid, and bile acid metabolism.

**TABLE 3:** Metabolites with ≥2 fold change at POST relative to PRE

Metabolite	Subpathway	Fold change	*p* value	*Q* value
tryptophan betaine	Tryptophan Metabolism	3.65	1.86E‐12	4.71E‐11
corticosterone	Corticosteroids	3.18	6.01E‐17	7.62E‐15
vanillic alcohol sulfate	Tyrosine Metabolism	3.08	5.95E‐06	1.67E‐05
isovalerylglycine	Leucine, Isoleucine and Valine Metabolism	2.55	2.66E‐14	1.13E‐12
cholate	Primary Bile Acid Metabolism	2.52	9.71E‐06	2.66E‐05
cholic acid glucuronide	Primary Bile Acid Metabolism	2.42	4.90E‐07	1.88E‐06
4‐methoxyphenol sulfate	Tyrosine Metabolism	2.33	1.50E‐08	9.77E‐08
3‐methylhistidine	Histidine Metabolism	2.16	8.54E‐08	3.97E‐07
10‐undecenoate (11:1n1)	Medium Chain Fatty Acid	−2.01	2.94E‐15	1.49E‐13
docosapentaenoate (n3 DPA; 22:5n3)	Long Chain Polyunsaturated Fatty Acid (n3 and n6)	−2.01	1.05E‐12	2.80E‐11
palmitate (16:0)	Long Chain Saturated Fatty Acid	−2.04	5.36E‐09	4.31E‐08
4‐methylhexanoylglutamine	Fatty Acid Metabolism (Acyl Glutamine)	−2.06	2.56E‐10	3.33E‐09
10‐nonadecenoate (19:1n9)	Long Chain Monounsaturated Fatty Acid	−2.13	4.09E‐10	4.93E‐09
(12 or 13)‐methylmyristate (a15:0 or i15:0)	Fatty Acid, Branched	−2.14	4.37E‐11	7.91E‐10
(2 or 3)‐decenoate (10:1n7 or n8)	Medium Chain Fatty Acid	−2.16	1.19E‐09	1.26E‐08
linolenate [alpha or gamma; (18:3n3 or 6)]	Long Chain Polyunsaturated Fatty Acid (n3 and n6)	−2.22	2.07E‐09	1.98E‐08
chenodeoxycholic acid sulfate (1)	Primary Bile Acid Metabolism	−2.25	6.88E‐08	3.29E‐07
pentadecanoate (15:0)	Long Chain Saturated Fatty Acid	−2.28	2.25E‐11	4.56E‐10
adrenate (22:4n6)	Long Chain Polyunsaturated Fatty Acid (n3 and n6)	−2.32	2.65E‐07	1.08E‐06
3‐carboxy‐4‐methyl‐5‐propyl‐2‐furanpropanoate (CMPF)	Fatty Acid, Dicarboxylate	−2.34	2.33E‐07	9.75E‐07
Acetoacetate	Ketone Bodies	−2.35	1.30E‐07	5.78E‐07
S‐methylmethionine	Methionine, Cysteine, SAM and Taurine Metabolism	−2.35	5.73E‐06	1.62E‐05
oleate/vaccenate (18:1)	Long Chain Monounsaturated Fatty Acid	−2.36	8.82E‐08	4.07E‐07
3‐hydroxybutyrate (BHBA)	Ketone Bodies	−2.36	1.57E‐08	1.01E‐07
10‐heptadecenoate (17:1n7)	Long Chain Monounsaturated Fatty Acid	−2.51	7.23E‐09	5.47E‐08
(14 or 15)‐methylpalmitate (a17:0 or i17:0)	Fatty Acid, Branched	−2.51	1.94E‐10	2.73E‐09
hexadecadienoate (16:2n6)	Long Chain Polyunsaturated Fatty Acid (n3 and n6)	−2.52	3.18E‐10	3.94E‐09
linoleate (18:2n6)	Long Chain Polyunsaturated Fatty Acid (n3 and n6)	−2.60	4.81E‐10	5.42E‐09
palmitoleate (16:1n7)	Long Chain Monounsaturated Fatty Acid	−2.67	1.42E‐08	9.37E‐08
tridecenedioate (C13:1‐DC)	Fatty Acid, Dicarboxylate	−2.68	4.11E‐16	2.97E‐14

*Note*: Mean fold change POST relative to PRE; *n* = 54 (52 males, 2 females); paired *t*‐test, *p* < 0.05; Benjamini–Hochberg method, *Q* < 0.10.

**FIGURE 3:**
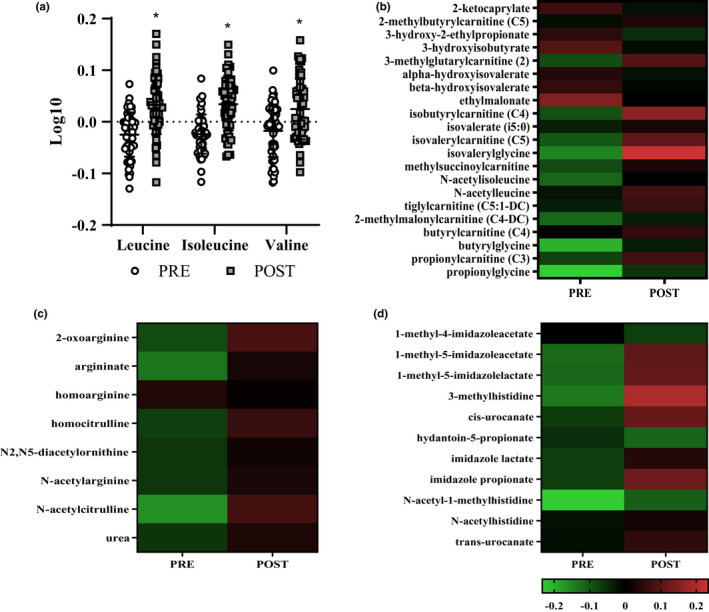
Mean ± SD log10 transformed branched‐chain amino acids (BCAA) PRE and POST IMT. *Different from PRE; paired *t*‐test *p* < 0.05, Benjamini‐Hochberg method, *Q* < 0.10 (a). Heatmap of mean log10 transformed BCAA metabolites (b), urea cycle metabolites (c), and histidine metabolites (d) that were significantly different at POST compared to PRE; paired *t*‐test, *p* < 0.05, Benjamini‐Hochberg method, *Q* < 0.10; *n* = 54 (52 males, 2 females).

The BCAAs leucine, isoleucine, and valine were all increased (*p* < 0.05, *Q* < 0.10) at POST compared to PRE (Figure 3a). Of the significantly different downstream BCAA metabolites, 71% were increased (*p* < 0.05, *Q* < 0.10) at POST compared to PRE (Figure 3b). There was an increase (*p* < 0.05, *Q* < 0.10) in 35% of urea cycle metabolites and 53% of histidine metabolites at POST compared to PRE (Figure 3c,d).

**FIGURE 4:**
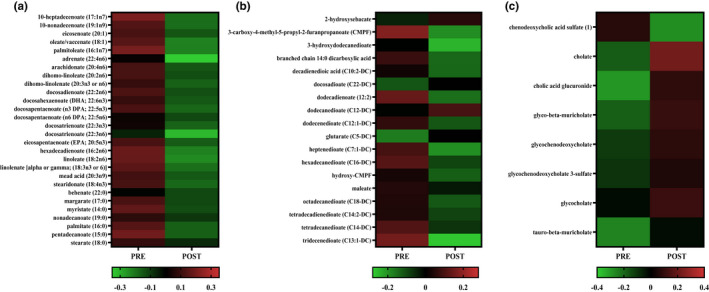
Heatmap of mean log10 transformed long chain fatty acid metabolites (a), fatty acid dicarboxylate metabolites (b), and primary bile acid metabolites (c) that were significantly different at POST compared to PRE; paired *t*‐test, *p* < 0.05, Benjamini–Hochberg method, *Q* < 0.10; *n* = 54 (52 males, 2 females).

At POST 93% of long chain fatty acid metabolites decreased (*p* < 0.05, *Q* < 0.10) compared to PRE (Figure 4a). There was a difference (*p* < 0.05, *Q* < 0.10) in 69% of dicarboxylate fatty acid metabolites, with four increased and 14 decreased at POST compared to PRE (Figure 4b). At POST, 58% of primary bile acid metabolites increased (*p* < 0.05, *Q* < 0.10) compared to PRE (Figure 4c).

## 5 DISCUSSION

The primary finding of this study was that 22‐week IMT resulted in large differences in the serum metabolome. This global metabolomics analysis identified that the majority of metabolites that changed during IMT were in sub‐pathways of amino acid and fatty acid metabolism. Increases in the BCAAs along with increases in downstream BCAA metabolites at POST compared to PRE IMT are supportive of greater endogenous BCAA availability which, concurrent with increases in circulating concentrations of total and free testosterone, likely facilitated the anabolic response of increased FFM during the training.

Following 22‐week IMT, FM decreased and FFM increased despite resulting in no net change in body mass. The positive body composition changes observed in the current study are consistent with previous investigations (Foulis et al., 2021; Margolis et al., 2012; Pasiakos et al., 2012; Williams, 2005). Given the effects of exercise training (Kraemer et al., 1988; Wilmore, 1996) alongside the modest negative shift in estimated energy balance and lack of change in estimated energy and protein intake in the current study, the positive changes in body composition are likely an adaptive response to the quantity and intensity of physical activity during the training. The observed changes indicate that the current US Army IMT environment promotes anabolism and supports positive adaptations to physical training in new military recruits independent of dietary intake. These findings are important due to the association between body composition and physical performance (Farina et al., 2021) as well as injury risk (Anderson et al., 2015; Jones et al., 2017) in various military populations.

Changes in the serum metabolome indicated that metabolites related to protein turnover and amino acid metabolism increased during 22‐week IMT. Greater FFM following physical training is facilitated, in part, by increased protein turnover (i.e., the combination of protein synthesis and protein breakdown) and concurrently upregulated amino acid metabolism (Biolo et al., 1995; Gibala, 2007; Tarnopolsky, 2004). The BCAAs, leucine, isoleucine, and valine, as well as their downstream metabolites, were all increased POST IMT. BCAAs both stimulate and serve as a substrate for muscle protein synthesis (Tom & Nair, 2006). The requisite upregulation in protein turnover for adaptation to physical training alongside no change in estimated protein intake suggests that the greater availability of circulating BCAA metabolites following IMT likely contributed to increases in FFM due to stimulation of protein synthesis and improvements in positive net protein balance (protein synthesis > protein breakdown) (Pasiakos & McClung, 2011). Indeed, higher concentrations of circulating BCAA metabolites POST IMT in the current study are in agreement with previous studies reporting an association with increased BCAAs and/or downstream metabolites with greater FFM (Jourdan et al., 2012; Margolis et al., 2012; Murphy et al., 2017). Similar to BCAA metabolites, increases in histidine, 3‐methylhistidine, and urea metabolites in the current study may indicate upregulated protein turnover (Holeček, 2020; Tipton et al., 2018). Both 3‐methylhistidine and urea are indirect markers of protein breakdown. However, in the absence of declines in FFM, increases in circulating histidine and urea metabolites likely reflect an upregulation in flux of amino acid metabolism due to protein breakdown for muscle remodeling and adaptation (Tipton et al., 2018). Specifically, increased urea cycle metabolites supports the metabolic disposal of amino group nitrogen derived from greater deamination of amino acids and reflective of BCAA flux during protein turnover. Collectively, increased BCAA, histidine, and urea cycle metabolites indicate overall greater protein turnover and likely reflect an adaptive response to physical activity stimuli contributing to gains in FFM during IMT.

Increases in both total and free circulating testosterone concentrations provide further evidence of an overall anabolic physiological response following IMT. Testosterone is a well‐characterized anabolic hormone and facilitates increased FFM as well as decreased FM (Bhasin et al., 2003; Katznelson et al., 1996). Accretion of FFM with elevated testosterone may be the result of enhanced efficiency of amino acid incorporation into newly synthesized proteins (Ferrando et al., 1998). Specifically, Ferrando et al. (1998) has reported that higher concentrations of circulating testosterone increase net protein synthesis through reutilization of endogenous amino acids in skeletal muscle. In the current study, concurrent elevations in circulating testosterone concentrations and BCAA metabolites may have synergistically contributed to an anabolic milieu to facilitate net positive protein balance to promote FFM accretion in response to 22‐week IMT.

Changes in the serum metabolome following 22‐weeks of IMT were predominately related to sub‐pathways of fatty acid metabolism. This finding is in agreement with previous investigations that have reported that fatty acid metabolites constitute the largest shift in the serum metabolome following exercise training (Pechlivanis et al., 2013; Sakaguchi et al., 2019; Schranner et al., 2020; Yan et al., 2009). In the current study, fatty acid metabolites with the largest changes from PRE to POST IMT were decreased and found within sub‐pathways of long chain fatty acid and dicarboxylate fatty acid metabolism. Both long‐chain and dicarboxylate fatty acid metabolites are important for energy generation through β‐oxidation and ω‐oxidation, respectively (Miura, 2013; Nakamura et al., 2014). Reductions in these metabolites are contrary to previous studies from our laboratory (Karl et al., 2017; Margolis et al., 2021) and others (Nieman et al., 2013; Stander et al., 2018), which reported increases in long‐chain and dicarboxylate fatty acid metabolites following acute aerobic exercise (4 h) or sustained military training (3 days). It is possible that declines in these metabolites may be the result of reductions in the relative dietary fat intake that were observed using an FFQ. However, though statistically different, the declines in relative fat intake were small (~6 g/1000 kcal/day) and may not account for the large shifts that were observed in fatty acid metabolites. More likely, changes in fat metabolites are related to the ~2 kg decrease in FM during IMT, with discrepancies between the past and current studies due to the state in which serum samples were collected and the study duration. In previous studies, increases in metabolites were reflective of a post aerobic exercise state (Karl et al., 2017; Margolis et al., 2021; Nieman et al., 2013; Stander et al., 2018), wherein increased concentrations of serum long‐chain and dicarboxylate fatty acid metabolites are likely reflective of increases in lipolysis to augment substrate availability for oxidation in skeletal muscle (Karl et al., 2017; Margolis et al., 2021). In the current study, serum was collected under resting fasted conditions and likely reflected longer‐term adaptation including reductions in total body FM. In agreement, previous investigations (Papandreou, Garcia‐Gavilan, Camacho‐Barcia, Hansen, Harrold, et al., 2021; Papandreou, Harrold, et al., 2021) have reported reductions in serum fatty acid metabolites and FM during weight loss. Others have demonstrated positive associations between serum long‐chain fatty acids and body fat (Papandreou, Garcia‐Gavilan, Camacho‐Barcia, Hansen, Sjodin, et al., 2021). Reductions in long‐chain fatty acid and dicarboxylate fatty acid metabolites in the current study were thus likely a metabolic signature of decreased FM and likely reflect increased reliance on endogenous fat stores for fuel during IMT.

While this study provides novel information on alterations in metabolomic profiles following IMT, several limitations should be noted. The lack of a complete dataset for dietary intake may have limited the current study's ability to detect differences between time points. We also recognize that the use of FFQ may limit the ability to interpret energy and macronutrient intake data, due to potential underreporting of absolute intakes (Bedard et al., 2004; Dahle et al., 2021). To correct for underreporting of absolute intake, FFQ data were analyzed as relative to 1000 kcal intakes. This method allows for assessment of the change in the pattern of energy and macronutrient intake. To fully understand the effects of absolute energy and macronutrients on changes in the serum metabolome during IMT, a more detailed method for assessing dietary intake, such as digital photography (McClung et al., 2018), should be used in future studies. Additionally, this study design does not permit the determination of the directionality of influence for the relationship between circulating BCAAs and FFM accretion. Thus, although the metabolic signatures previously discussed support a moderating effect of circulating BCAAs on FFM accretion, the directionality of this relationship is undetectable without targeted kinetic tracer methods. Lastly, while the current study included both male and female participants, only 2 females completed the study. As responses to IMT have been reported to differ by sex (Hennigar et al., 2021; Margolis et al., 2012), alterations in the serum metabolome during IMT likely differ between men and women following IMT. As such, further investigation is warranted to understand the metabolomic responses of women during IMT (Wardle et al., 2021).

In conclusion, results from the current study indicate robust changes in the serum metabolome following 22‐weeks of IMT. These changes were most evident within pathways of amino acid and fatty acid metabolism. Findings from this study give new insight into the anabolic response to IMT by providing evidence that increases in endogenous BCAAs in combination with increased circulating total and free testosterone may support accretion of FFM during IMT.

## AUTHOR CONTRIBUTIONS

Lee M. Margolis, James P. McClung, and J. Philip Karl conceived and designed the research; Jess A. Gwin, Adrienne Hatch‐McChesney, Kenneth P. Pitts, Anthony J. Karis, Christopher T. Carrigan, J. Philip Karl, and Lee M. Margolis performed the experiments; Jess A. Gwin, Adrienne Hatch‐McChesney, and Lee M. Margolis analyzed the data; Jess A. Gwin, J. Philip Karl, and Lee M. Margolis interpreted the experiment results; Jess A. Gwin and Lee M. Margolis prepared the figures; Jess A. Gwin and Lee M. Margolis drafted the manuscript; Jess A. Gwin, Adrienne Hatch‐McChesney, Kenneth P. Pitts, Rory P. O'’Brien, Anthony J. Karis, Christopher T. Carrigan, James P. McClung, J. Philip Karl, and Lee M. Margolis edited and revised the manuscript; Jess A. Gwin, Adrienne Hatch‐McChesney, Kenneth P. Pitts, Rory P. O'’Brien, Anthony J. Karis, Christopher T. Carrigan, James P. McClung, J. Philip Karl, and Lee M. Margolis approved final version of the manuscript.

## FUNDING INFORMATION

This material is based on the work supported by the US Army Medical Research and Development Command.

## CONFLICT OF INTEREST

The authors declare that they have no conflicts of interest relevant to the content of this article. The opinions or assertions contained herein are the private views of the authors and are not to be construed as official or as reflecting the views of the Army or the Department of Defense. Any citations of commercial organizations and trade names in this report do not constitute an official Department of the Army endorsement of approval of the products or services of these organizations.

## ETHICS STATEMENT

This study was approved by the U.S. Army Medical Research and Development Command Institutional Review Board and adhered to the policies for protection of human subjects as prescribed in the U.S. Department of Defense Instruction 3216.02, and the research was conducted in adherence with the provisions of 32 CFR Part 219.
